# Surviving the Antarctic Winter—Life Stage Cold Tolerance and Ice Entrapment Survival in The Invasive Chironomid Midge *Eretmoptera murphyi*

**DOI:** 10.3390/insects11030147

**Published:** 2020-02-26

**Authors:** Jesamine C. Bartlett, Peter Convey, Scott A. L. Hayward

**Affiliations:** 1 School of Biosciences, University of Birmingham, Edgbaston B15 2TT, UK; jesamine.bartlett@nina.no; 2 Norwegian Institute for Nature Research, Høgskoleringen 9, 7034 Trondheim, Norway; 3 British Antarctic Survey, NERC, High Cross, Madingley Rd, Cambridge CB3 0ET, UK; pcon@bas.ac.uk

**Keywords:** microclimate, insect physiology, overwintering, invasion biology, thermal thresholds

## Abstract

An insect’s ability to tolerate winter conditions is a critical determinant of its success. This is true for both native and invasive species, and especially so in harsh polar environments. The midge *Eretmoptera murphyi* (Diptera, Chironomidae) is invasive to maritime Antarctic Signy Island, and the ability of fourth instar larvae to tolerate freezing is hypothesized to allow the species to extend its range further south. However, no detailed assessment of stress tolerance in any other life stage has yet been conducted. Here, we report that, although larvae, pupae and adults all have supercooling points (SCPs) of around −5 °C, only the larvae are freeze-tolerant, and that cold-hardiness increases with larval maturity. Eggs are freeze-avoiding and have an SCP of around −17 °C. At −3.34 °C, the CT_min_ activity thresholds of adults are close to their SCP of −5 °C, and they are likely chill-susceptible. Larvae could not withstand the anoxic conditions of ice entrapment or submergence in water beyond 28 d. The data obtained here indicate that the cold-tolerance characteristics of this invasive midge would permit it to colonize areas further south, including much of the western coast of the Antarctic Peninsula.

## 1. Introduction

Survival in the polar regions requires the ability to tolerate conditions that regularly fall below freezing, sometimes for months at a time. As poikilothermic ectotherms, terrestrial invertebrates do not generally have the ability to thermoregulate and are particularly vulnerable to such temperatures. Antarctic winters are long and harsh, and even in the warmer maritime Antarctic where two species of insects are native, temperatures remain below freezing for several months of the year [1]. These insects, like any terrestrial invertebrate that lives in the polar regions, are at risk of their body fluids freezing, causing potentially fatal injury [2]. To survive such conditions, invertebrates have developed physiological and behavioral cold-tolerance strategies that allow them to either tolerate freezing or avoid it [3]. These cold-tolerance adaptations will also determine the success of any ingressing or introduced species to the polar regions. Therefore, understanding how an invasive species survives polar winter conditions is crucial in understanding its potential to establish and spread.

Convey et al. [1] highlight the importance of understanding the heterogeneous features of polar soils at small and biologically relevant physical scales and how this influences patterns in microhabitat temperatures experienced by soil biota. Terrestrial invertebrates in these microhabitats may also be vulnerable to flooding and ice entrapment, resulting from snow melt in the summer and freezing in the winter, especially in soils and vegetation underlain by permafrost. This creates a challenging environment where invertebrates are at risk of low temperature stress, as well as ice entrapment and associated anoxic conditions [4]. Foraging, mating and overall movement are also affected by low temperatures; the point at which an invertebrate loses neuromuscular coordination is defined as the critical thermal minimum (CT_min_) and, if temperatures continue to decline beyond this, then movement will cease altogether, and the invertebrate will enter a ”chill coma” [5]. To mitigate these risks, some chironomids will migrate to find suitable microhabitats [6]. Similar behavior is seen in Antarctic Collembola and Acari, which seek thermally preferential microhabitats and, in the instance of the mite *Alaskozetes antarcticus*, will choose a lower temperature microhabitat in order to maintain a cold-hardened state [7].

As mean monthly temperatures in Antarctica remain below 0 °C for many months, even with snow cover buffering temperature extremes [1,8], soil invertebrates must deal with the threat of freezing temperatures. To do this, they use one of two general strategies: freeze-tolerance or freeze-avoidance [9,10]. Freeze-avoiding species, such as the native Antarctic springtail *Crytopygus antarcticus* and the mite *Alaskozetes antarcticus* [7], cannot survive internal ice formation but can survive sudden cold shocks and low temperatures that remain above their supercooling point (SCP) [9]. The SCP of an organism is the temperature at which ice formation starts to occur in its body, with SCPs being stable within an individual but variable to a degree within a population [11]. Broadly, where the SCP is below the lower lethal temperature (LLT), the species is classed as freeze-avoiding. In the alternative strategy, freeze-tolerance, individuals can survive the formation of ice in body fluids (but not generally within cells) via a suite of mechanisms [12,13]. The LLT of freeze-tolerant species is typically below their SCP. Relatively few species of Antarctic-region terrestrial invertebrates are freezing tolerant [14], but one insect example is provided by fourth instar larvae of the endemic sub-Antarctic and invasive maritime Antarctic midge, *Eretmoptera murphyi* [15]. 

**Figure 1:**
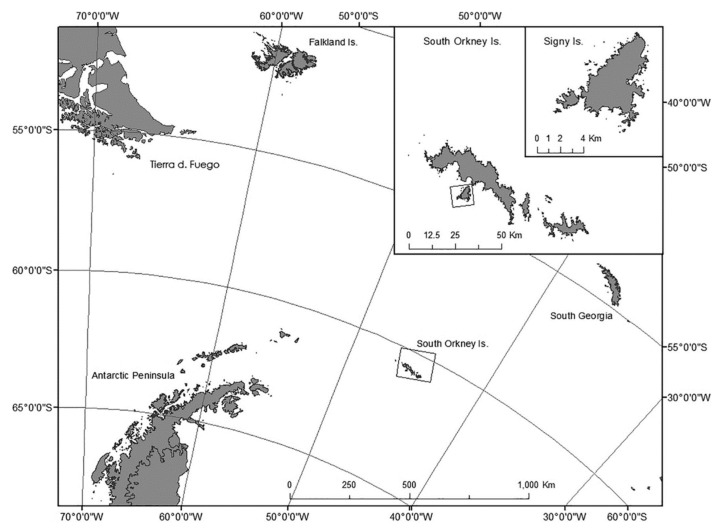
Location of Signy Island in the South Orkney Island archipelago.

*Eretmoptera murphyi* is thought to have been introduced to Signy Island in the maritime Antarctic (60 °S, 45°W) (Figure 1) in the 1960s during a plant transplant experiment [14]. The midge is native to sub-Antarctic South Georgia (55°S, 45°W) (Figure 1), where it is paleoendemic [15,16]. It has a life cycle of two years, spending most of that as one of four larval instars [17]. In addition to being freeze-tolerant, this species has several other pre-adaptations, enabling survival of conditions experienced in the maritime Antarctic, including desiccation tolerance [18], and the ability to rapidly cold-harden [19]. As *E. murphyi* is resident in potentially waterlogged moss banks, the risk of summer flooding and winter ice-entrapment may result in oxygen-depleted habitats that may persist for months [4,20]. *Eretmoptera murphyi’s* ability to respire underwater and, for some individuals, to survive beyond 28 days of water submergence and ice entrapment imply a remarkably high level of anoxia tolerance [21]. However, importantly, these previous assessments were conducted using tap water, and the species’ ability to survive an entire winter entrapped in ice under more field-realistic conditions, where temperatures will not rise above freezing for months [22,23], remains unknown. 

As with studies of the related *Belgica antarctica* [24,25,26], most physiological examinations of *E. murphyi* have been conducted on mature larvae—with the exception of two studies that examined desiccation and heat tolerance in eggs [27,28]. Thus, knowledge of the cold-tolerance abilities of different life stages remains limited. Differences in cold tolerance between life stages of an invasive insect can be a particularly important factor in assessing their range potential in environmental niche models [29], particularly for insects that may overwinter in more than one life stage [3]. Enhanced cold tolerance is common in the larval stages of Chironimidae and is typically limited in adults [30,31,32,33]. However, to date, few studies have compared cold tolerance between the larval instars or different life stages of a single chironomid species. Studies of other dipterans have reported a range of tolerances across larval, pupal and adult stages within the same species [34,35]. Variation in the levels of cold tolerance between different larval instars have also been found in moths, e.g., *Epiphyas postvittana* [36] and *Spilonota ocellana* [37], as well as in mites [29].

As the predicted warming of the polar regions gains pace [38], more species are expected to be able to colonize higher latitude environments [39], and predicting which species are able to cope with the challenging environment is essential to understanding their invasion ecology e.g., [40,41]. A successful species in the polar regions must be able to develop, feed and reproduce in the short available growth seasons [42,43], as well as be able to overwinter successfully [13,44,45]. In the last few decades, there has been a move to highlight the importance of ecologically relevant assessments of a species’ thermal tolerance e.g., [3,46], both to accurately reflect drivers of thermal physiology for species of interest but also as a key factor in establishing the risk of a species becoming invasive. It is insufficient to establish the boundaries of lethal temperatures and broad cold-tolerance strategies alone. Rather, the duration, intensity and pattern of cold stress exposure must also be considered [47], and experimental assessments should be relevant to the conditions experienced in natural field conditions by the species in question [48].

This study examines the cold-tolerance abilities and strategies of all life stages of the invasive Antarctic midge, *E. murphyi*. Building on the work of Everatt et al. [49], we also assess the ability of larvae to survive prolonged periods of ice entrapment and associated anoxia tolerance and, concurrently, investigate the ability of the midge to survive more than 28 days underwater. Results are placed in the context of winter microclimate data relevant to *E. murphyi*’s habitats on Signy Island, and this species’ invasive potential further south is discussed.

## 2. Materials and Methods

### 2.1. Study Site and Sample Collection

All experiments, apart from those on ice-entrapment and water submergence, were conducted in laboratories at the British Antarctic Survey’s Signy Island Research Station, South Orkney Islands, maritime Antarctic (Figure 1), during the 2016/2017 austral summer season. Samples were collected as described by Bartlett et al. [17]. All eggs used were confirmed to be at the first (opal) developmental stage [17] using a dissecting microscope (Leica EZ4). If any eggs showed signs of yellowing or embryonic development, the entire egg sac was discarded. Individual eggs were removed from the egg sacs through microscopic dissection, with care taken to avoid damage. Other live *E. murphyi* samples used in the ice-entrapment and water submergence experiments were collected during the 2014/15 austral summer by BAS station staff on Signy Island and were returned to the United Kingdom by ship in refrigerated (+4—5 °C) cold storage (10 weeks) and then maintained on their native substrate at +5 °C at the University of Birmingham until use in late 2015 and early 2016.

### 2.2. Overwintering Environmental Data

In order to determine winter microhabitat conditions on Signy Island, three temperature loggers (Tinytag Plus II TGP-4500, Gemini Data Loggers, Chichester, UK) were placed below the ground surface within the top 5 cm of the soil profile, which is where the larvae are known to reside [17]. This site was ~10 m a.s.l. behind the research station in a moss bank. The loggers were programmed to collect data from 12 March 2017 until February 2018, recording every 2 h with a manufacturer’s statement of accuracy ± 0.01 °C (Gemini data sheet, 2018). Only data collected prior to 22 January 2018 were used due to faults encountered with data loggers. Total recording time was therefore from 1 March 2017 to 21 January 2018 (inclusive).

### 2.3. Cold-Tolerance Ability of E. Murphyi

#### 2.3.1. Measurement of Supercooling Points

SCPs were assessed for each life stage as they became available: adults (*n* = 20); pupae (*n* = 6); all four larval instars (L1, *n* = 22; L2, *n* = 22; L3, *n* = 23 and L4, *n* = 22); eggs (*n* = 15) and the entire lipid egg sac (*n* = 32). Some adults (*n* = 5) were obtained from field-collected pupae that enclosed in the laboratory; the remainder (*n* = 15) were obtained direct from the field. These were recorded separately, and their SCPs noted, to determine whether laboratory-raised adults generated different results from field-collected adults. The SCPs of individuals of each life stage were determined by cooling slowly from +5 °C to −25 °C at 0.2 °C min^−1^ in an alcohol bath (Haake Phoenix II C50P, Thermo Electron Corporation, Karlsruhe, Germany) [19]. Each individual was placed in contact with a thermocouple using Oecotak in the following groupings: *n* = 1 per thermocouple for egg sacs, adults, pupae, L4 and L3 larvae; *n* = 2 for L2; *n* = 5 for L1 and *n* = 10 for individual eggs. Thermocouples were placed within an Eppendorf tube and inserted into the bottom of a test tube that was two-thirds submerged in the alcohol bath cooling fluid. The temperature of the individuals was recorded using Picolog Recorder Software and a TC-08 multichannel data logger (Pico Technology Limited, St Neots, UK), and the SCP was defined as the onset of the freezing exotherm.

#### 2.3.2. Cold-Tolerance Strategy of Juvenile Life Stages

The cold-tolerance strategy (either freeze-tolerant or freeze-avoiding) of all juvenile life stages was assessed by exposing them to temperatures of −5, −10, −15, −20, −25 or −30 °C, with a +5 °C control. Temperature was reduced from the control temperature of +5 °C to the target temperature at a rate of 0.2 °C min^−1^, and once the target temperature was reached, samples were removed immediately. For each temperature exposure, four replicates of five individual larvae (*n* = 20), and ten replicates of a single egg sac, were placed in a sealed Eppendorf tube with a thermocouple wire threaded through a small hole in the lid. The 10 egg sacs contained *c.* 630 eggs between them (based on Bartlett et al. [27]). After each treatment, individuals were removed and placed in a petri dish containing moist Signy soil substrate and kept at control conditions in a dark refrigerator. Larval survival was determined 72 h after exposure by assessing movement or reaction to stimulation from a fine paintbrush. Egg survival was assessed after 35 d by dissecting egg sacs and determining the proportion of hatched vs. unhatched eggs.

#### 2.3.3. Lower Thermal Activity Thresholds of Adults

Adult *E. murphyi* were chosen for lower thermal activity threshold assessment as they are the most mobile life stage. All adults were field-collected and kept at +5 °C for 24 h prior to experimentation. These experiments were conducted in an aluminum block arena [see 49], the temperature of which was regulated using an alcohol bath (Haake Phoenix II C50P, Thermo Electron Corporation, Karlsruhe, Germany). A thermocouple wire attached to a digital thermometer was inserted into the arena in order to monitor and record the temperature experienced. Adult activity was monitored using a digital video camera with a macro lens (Infinity 2, Teledyne Lumnera, Ottawa, Canada). Six individuals were placed within the +5 °C arena for 1 h before recording to allow acclimation. The video and temperature data were captured using Studio Capture DT software (Studio86Designs, Lutterworth, UK) as the temperature was reduced from +5 °C to −20 °C at a rate of 0.2 °C min^−1^ [5]. The temperatures at which each individual last performed a coordinated movement (CT_min_) and the final involuntary movement of either legs or antenna (entry into chill coma) were recorded.

### 2.4. Overwintering in Ice and Prolonged Submergence in Water

#### 2.4.1. Field vs. Laboratory Water

In order to first ascertain the experimental impact of using different water types in freezing experiments [21], we measured the freezing point of 18 MΩcm deionized water (DIW) and Signy field water (FW). Signy field water was prepared as described by Bartlett et al. [17,27]. Seven 1.5 mL Eppendorf tubes containing each water type were placed in an alcohol bath (as above) and taken from +5 °C to −10°C at a rate of 0.2 °C min^−1^, and the freezing point was recorded.

Three groups of *n* = 5 L4 larvae were placed in either DIW or FW and exposed to −3 °C for either 1, 3 or 7 d. Upon the ending of each experiment, it was noted that ice formation did not occur in DIW treatments. Survival was assessed 72 h post-exposure, as previously described.

#### 2.4.2. Long-Term Ice Entrapment

The ability to survive extended periods of ice-entrapment/anoxia was assessed using L4 larvae entrapped in FW ice for 28, 42 or 63 d at −3 °C. Larvae were either nonacclimated (NA)—taken directly from storage conditions (+5 °C in soil)—or winter-acclimated (”A”) by keeping samples at 0 °C in soil for one week prior to ice entrapment. Two controls were used: control 1 (”C1”) tested the effect of submergence in FW at +5 °C for the entire duration of the experiment and control 2 (”C2”) were maintained at +5 °C in soil with no submergence stress. In all instances, three groups of *n* = 10 larvae were used for each time point. Survival after treatment (including controls) was assessed every 48 h for a further 25 d post-treatment, taking post-treatment assessment to 28 days total. All experiments were conducted in the dark in a bench freezer (Fryka B3-20) set to either +5 °C or −3 °C. The freezer unit was calibrated using digital thermometers, and the temperature throughout the experiment was recorded using a data logger (Tinytag Transit, Gemini Data Loggers, Chichester, UK). Due to the length of the post-exposure analysis, soil used in the control and recovery conditions was initially prepared by baking at 60 °C for 24 h in order to inhibit any fungi that may have been present.

### 2.5. Statistical Analysis

All data were tested for normality prior to further analysis using a Shapiro-Wilk test (alpha = 0.05), and transformed if necessary. The quality control assessments of the effects of field vs. laboratory waters were analyzed with *t*-tests. Cold-tolerance data was Log10-transformed to fit the assumptions of parametric testing, and analyzed with a two-way ANOVA with Tukey’s multiple comparisons (column effects). All further data were nonparametric and were analyzed with Mann-Whitney U or Kruskal-Wallis tests, followed by Dunn’s multiple comparisons where appropriate. Ice entrapment data were first analyzed and plotted with a least-squares linear regression [50], then assessed for multiple comparisons with a Kruskal-Wallis test with Dunn’s multiple comparisons.

## 3. Results

### 3.1. Supercooling and Activity Thresholds

**Figure 2:**
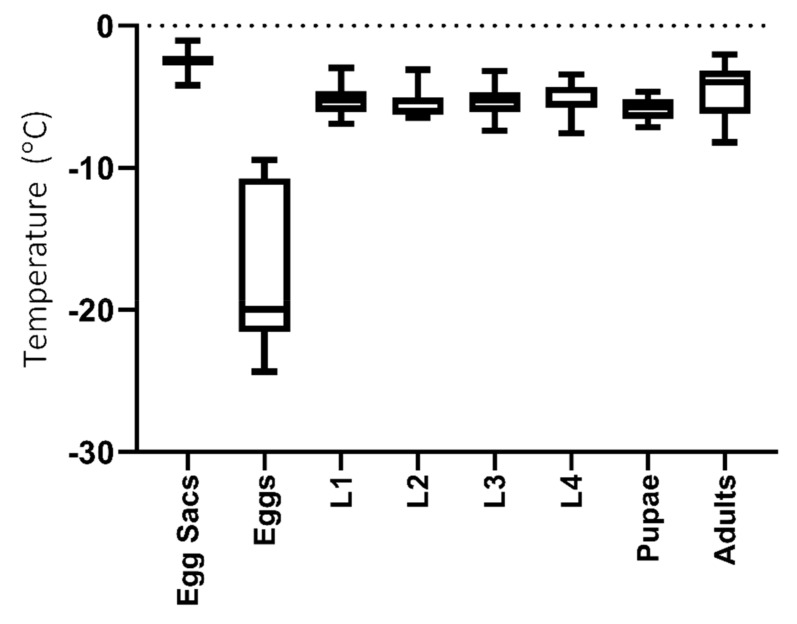
Mean (± SEM) supercooling points (SCP) of different *E. murphyi* life stages. Egg sacs, *n* = 32; individual eggs, *n* = 15; pupae, *n* = 6; adults, *n* = 20 and larval instars: L1, *n* = 22; L2, *n* = 22; L3, *n* = 23 and L4, *n* = 22.

There was no difference in the SCPs measured in the four larval instars and pupae (Kruskal-Wallis; *H* = 6.6, *p* = 0.24, *n* = 115), with mean values ranging between −5.25 °C (L1 and L4), −5.59 °C (L2) and −5.81 °C (pupae). The egg sac matrix had an SCP of −2.45 ± 0.12 °C SEM (*n* = 32), whilst individual eggs had an SCP of −17.58 ±1.37 °C SEM (*n* = 15) (Figure 2). 

**Figure 3:**
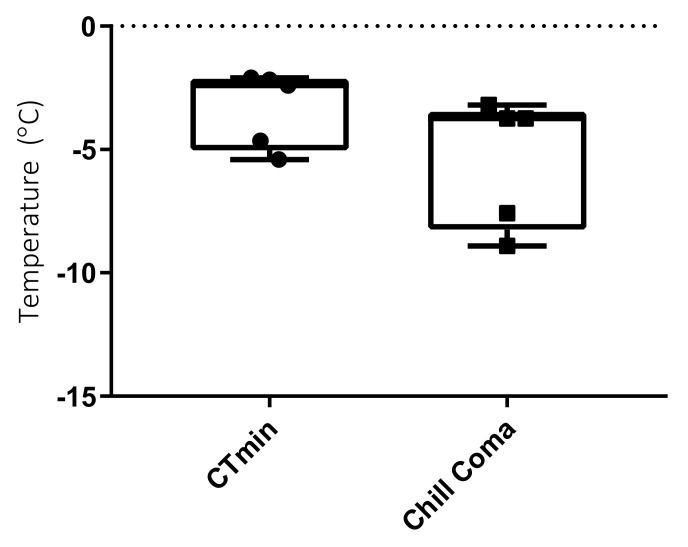
Mean adult activity thresholds ± SEM (*n* = 5 for both samples). CT_min_ represents loss of coordinated movement, and chill coma represents last involuntary movement.

Adults had an SCP of −5.07 ± 0.6 °C SEM (*n* = 20), with no difference between the SCPs of ”lab” vs. ”field” adults (Mann Whitney U = 23, *p* = 0.23, *n* = 5 ”lab” and *n* = 15 ”field”). Mean adult *E. murphyi* CT_min_ was -3.34 ± 0.7 °C (*n* = 6), whilst the chill coma was -5.44 ± 1.17 °C SEM (*n* = 5) (Figure 3)

### 3.2. Cold-Tolerance Strategy of Different Life Stages

**Figure 4:**
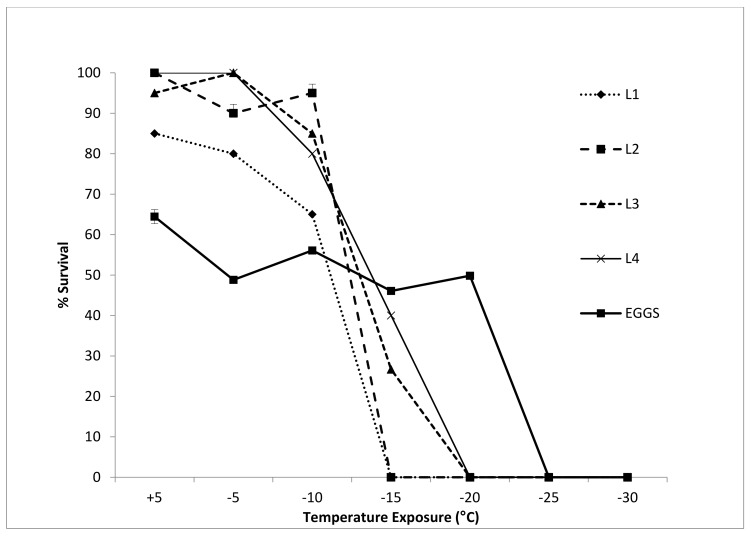
Mean survival ± 95% CI survival of *E. murphyi* larval instars (L1–L4; *n* = 20 for each instar) and eggs (*n* = 630 individual eggs from *n* = 10 egg sacs) after exposure to declining temperatures (discrete treatments). Larval survival was determined 72 h after exposure. Egg survival was assessed after 35 d by determining hatching success.

Survival amongst all life stages declined significantly with temperature (two-way ANOVA *F* (6,24), *p* < 0.0001). Differences between life stages at each temperature treatment, compared to the control, were largely driven by the response of eggs to cooling compared to the larval instars (Table 1): Early larval instars (L1 and L2) reached 100% mortality between −10 °C and −15 °C. This occurred between −15 and −20 °C for L3 and L4 larvae and between -20 and −25 °C for eggs (Figure 4).

### 3.3. Overwintering in Ice and Prolonged Submergence

#### 3.3.1. Field vs. Laboratory Water Freezing Point 

FW had an SCP of −3.06 ± 0.1 °C, significantly higher than that of DIW at −4.31 ± 0.5 °C (unpaired *t*-test; *t_12_* = 3.9, *p* = 0.002). DIW did not freeze, resulting in lower survivals in FW after 7 d, as ice entrapment had not been experienced in the DIW condition (unpaired *t*-test (4); *t* = 5.5, *p* = 0.005). 

#### 3.3.2. Prolonged Ice Entrapment and Submergence

**Figure 5:**
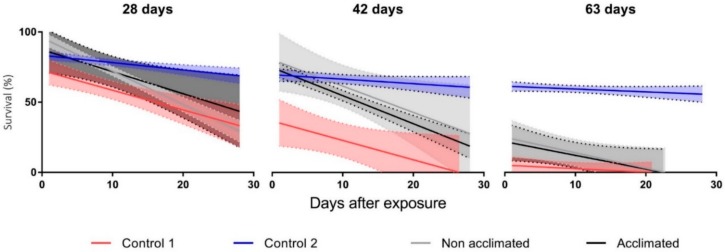
Long-term survival of fourth instar *E. murphyi* larvae assessed every 48 h over 28 d post-treatment following exposure to 28, 42 or 63 days entrapped in ice at −3 °C. Control 1 = +5 °C submerged in field water. Control 2 = +5 °C soil control. Winter ”acclimated” samples were kept at 0 °C and ”non acclimated” were maintained at +5 °C in soil conditions for one week prior to exposure treatment. N = 10 × 3 replicates.

Overall, only after 63-d exposure did survival across all the treatments differ significantly from the +5 °C soil control (Kruskal-Wallis test, overall interaction of conditions with control: 28 d, H = 5.5, *p* = 0.13; 42 d, H = 13.2, *p* = 0.004 and 63 d, H = 18.3, *p* < 0.001) (see Table 2 for multiple comparisons). At 42 d, there was only an overall difference between the submergence treatment and the soil control, whilst at 28 d, there were no differences between any of the treatments or the soil control (Table 2). Figure 5 shows how survival declined during the 28-day survival assessment period following each ice entrapment treatment. For all 42 and 63-day treatments, except C2, survival declined to 0% during this 28-day monitoring period.

### 3.4. Overwintering Environmental Data

**Figure 6:**
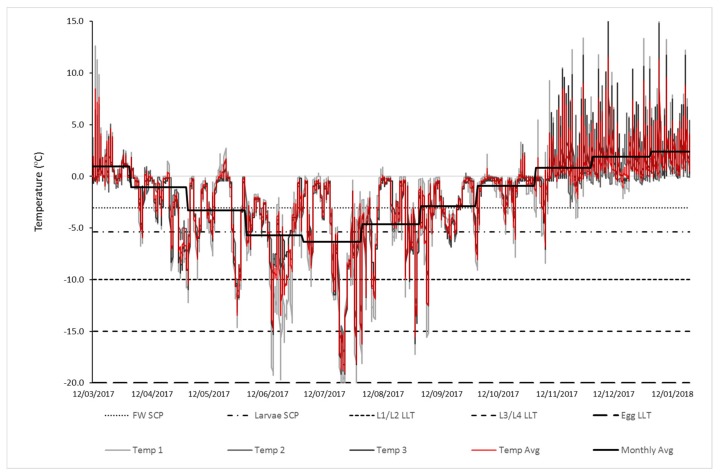
Soil temperature (within top 5 cm) on Signy Island from 12 March 2017–21 Jan 2018. Individual data loggers (Temp 1–3), the average between loggers (Temp Avg) and monthly average are shown. Mean SCP for field water and *E. murphyi* larval instars are highlighted, as well as mean lower lethal temperatures (LLTs) for different life stages.

Below-ground (top 5 cm soil) monthly temperatures in the period March 2017 to January 2018 averaged between +2.39 ± 0.09 °C SEM in January and −6.32 ± 0.16 °C SEM in July. The coldest temperature recorded was −20.27 °C on 23 July 2017 in the middle of the coldest period recorded in the dataset—four consecutive days below −10 °C between 20 and 23 July 2017. The warmest temperature recorded was +16.9 °C on 9 December 2017 (Figure 6). Seven months had mean temperatures below 0 °C, four of which were below the SCP for field water (−3.05 °C), with two months below the SCP for all *E. murphyi* larval instars (−5.35 °C). The LLT for eggs (−20 °C) was never reached; however, the LLT of L3/L4 was met for a total of 49 h over a 7-day period (20–26 July). The longest period at temperatures below L3/L4 LLT was 37 consecutive hours between 20 and 23 July 2017. L1/L2 LLTs were exceeded for 154 h over 28 d. The longest period spent at or below L1/L2 LLTs was 25 h between 26 and 31 July 2017. 

## 4. Discussion

Polar regions are characterized by air temperatures below 0 °C for much of the year and land covered by snow and ice [51]. Yet, within the top layers of subsurface soil, temperatures experienced by resident flora and fauna may differ from the air temperature, as factors such as vegetation and snow cover will provide insulation [1,8]. Previous microhabitat assessments on Signy Island have recorded a minimum below-ground temperature of −14.8 °C in July 1987 [8]. This was recorded 3 cm below the surface at a site ~10 m a.s.l. and in a similar location to the current study. A minimum surface temperature of −17.1 °C has also been recorded at this site [8]. The minimum below-ground temperature reached during the current study was −20.27 °C in July 2017, and in the middle of the coldest period recorded in the dataset, four consecutive days were below −10 °C. It is worth noting that Convey et al. [1] also reported microhabitat temperatures on Signy in recent years but at 150 m a.s.l. at Jane Col—an exposed hill site. They recorded a minimum winter temperature in 2009 of −8.7 °C, considerably warmer than both this study and that of Davey et al. [8] despite a minimum air temperature of −30.1 °C recorded the same year. Much of this variation may be the result of snow cover, as the effect of snow as an insulator is well-documented at micro and macro scales [1,52,53]. At the time of the Davey et al. [8] recorded minimum, snow cover was 6 cm, whilst Jane Col is usually covered by deep (> 0.5 m) snow drifts between June and November [54], which would explain the warmer temperatures reported by Convey et al. [1]. Snow cover depth was not recorded as part of this study but may be a valuable consideration for future assessments of field-relevant physiological assessments. 

Data in the current study were collected from three sites within 3 m of one another. As *E. murphyi* has a distribution of at least 80,000 m^2^ over an undulating landscape [55], the environmental conditions described here may not be representative of the entire distribution range due to likely variation in snow depth, as exemplified by the difference in minimum temperatures between this study and that of Davey et al. [8], despite the same locale. It is worth noting, however, that the location of the temperature loggers in this study is associated with high *E. murphyi* abundance [55]; thus, prior conditions are not prohibitive to survival. 

The most striking difference in the cold-tolerance strategies of the life stages of *E. murphyi* was the ability of eggs to supercool to temperatures of −17.58 °C, compared to larval instars, pupae and adults, which all had SCPs around −5 to −6 °C. Previous studies had identified that L4 larvae are freeze-tolerant [13,19], and the current study has now confirmed that all larval instars can tolerate freezing (Figure 4). However, tolerance of temperatures below the SCP is not consistent across all instars and appears to increase as larvae mature. Eggs had both a low SCP and good survival down to temperatures very close to their SCP, suggesting that they are freeze-avoiding [9]. Indeed, eggs showed better survival at -15 and −20 °C than all other life stages, with 100% mortality not being reached until temperatures between −20 and −25 °C (Figure 4). The spread of SCP values for eggs, some well below −20 °C (Figure 2), helps explain this level of measured cold tolerance. Despite this, the midge is not thought to overwinter in the egg stage [17,28,56]. High cold tolerance in the egg stages are seen across insect groups both in Antarctica [53] and globally, with many species demonstrating lower egg SCP than their other life stages (e.g., temperate and subtropical Diptera (Ceratopogonidae) [57], North American Lepidoptera [58], Fennoscandinavian Lepidoptera [59] and temperate Hemiptera [60]). This level of cold tolerance in eggs is therefore not unique and may be the consequence of egg characteristics, such as higher fat content and sclerotization of the eggshell providing a physical barrier to nucleators, which is found in other Nematocera [61], the sub-order which includes Chironomidae, or may be an adaptive trait that allows flexibility for potential overwintering, as is seen in some Hemiptera species [62]. 

Environmental data presented here suggest that, if necessary, eggs could survive the temperatures experienced in the soil in winter on Signy. Based on these data, it may not be possible for some larvae to survive winter, however, particularly L1 and L2 instars (compare Figure 4 and Figure 6). That said, *E. murphyi* larvae are capable of rapid cold hardening (RCH), during which they can lower their LLT by up 6.5 °C for juvenile larvae and 2.5 °C for mature larvae [19]. Worland [13] also reported that mature larvae could survive temperatures as low as −20 °C after a 4-d acclimation at −4 °C. In both studies, it was considered that acclimation to such low temperatures was unnecessary, as Signy winter conditions were thought to be milder. However, our data indicate that acclimation may be a necessary process in some microhabitats as a result of variation in snow cover. Based on the data obtained in the current study, a decrease in LLT of 6.5 °C for L1/L2 larvae through RCH would reduce it to −16.5 °C. This would reduce their exposure to the LLT to just 31 h over 5 days in July, compared to 154 h over several months. For mature larvae, the lower LLT would similarly move from −15 °C to −17.5 °C, reducing exposure to LLT from 49 h over two months to 20 h over just 4 d. 

It has previously been suggested that the L2 and L4 instars are the only stages of *E. murphyi* that overwinter [17,22,62]. However, considering the SCP, LLT and acclimation potential of the larvae, all instars appear to have the physiological capacity to overwinter, as is seen in the closely related *B. antarctica* [63,64]. In order to verify this, further studies on the acclimation capabilities of early instars, the long-term freeze tolerance of all instars at temperatures relevant to Signy and confirmation of which instars overwinter are required. Pupae are not thought to overwinter [19], and their ability to tolerate cold remains untested. However, with SCPs similar to larvae (−5.81 ± 0.36 °C), there is little risk to pupal survival during the summer months when they occur. 

Adult CT_min_ and chill coma values were both close to their SCP, around −5 °C. Given chilling injury begins soon after chill coma [65], this life stage is likely the most vulnerable to temperature and is perhaps best classified as chill-susceptible [9]. Whilst the mean SCP for adults was in line with that of larvae and pupae, there was one individual with an SCP of −14°C. Both the CT_min_ and the onset of chill coma for adults could suggest a potential bimodality, with only some individuals entering chill coma below the mean SCP, but this is likely a result of the variability within individual adult SCP. Regrettably, only five individual adults were available for CT_min_ and chill coma assessment; thus, a larger sample size would improve our understanding of this life stage. A study on *E. murphyi’s* closest relative, *B. antarctica*, found that adults of this species were also freeze-intolerant and were unable to rapidly cold-harden to temperatures below −5 °C [66]. The lack of cold-hardiness in adults of both species is perhaps unsurprising as neither survives long in adult form during summer months, generally emerging and only being active on warm days [17,63,64]. 

The physiological mechanisms involved in *E. murphyi* cold tolerance are likely to be governed by similar mechanisms to that of *B. Antarctica*, which has recently been found to draw heavily on glycogen reserves in response to cold exposures [67]. The resulting glucose mobilization is well documented to act as a cryoprotectant and aid cold tolerance [68]. Lipids may also play a role, with these reserves drawn on during repeated cold exposures by *B. Antarctica* [69]. For *E. murphyi*, the difference in energy stores between larval instars may explain the increasing level of cold tolerance seen with larval maturity. Studies into *B. antarctica* have also found that the upregulation of heat shock proteins, such as *hsp60* and *90*, with a freeze event indicates they are biomarkers of freezing stress and associated sublethal protein damage that could affect long-term health post-exposure. 

The winter microclimate data obtained here indicate that soil conditions on Signy can be below the SCP of field water for a maximum of four months (May-Aug) (Figure 6). It is likely, therefore, that many *E. murphyi* habitats will be frozen for this time. Whether this extends to complete ice entrapment of the larvae for the entire duration is unknown, as this would rely on a prior flood event or very high levels of substrate water saturation immediately prior to the freeze event. The data presented here suggest that L4 larvae can survive for an extended period following ice entrapment of up to 28 days. Long-term survival following 42 days of ice entrapment, however, declined to around 10%. No individuals survived much beyond a further 20 d after 63 days of ice entrapment (Figure 5). This suggests prolonged anoxia and chilling results in irreparable injuries that will impact survival over weeks, rather than days, post-exposure. Typically, survival after a stress treatment is measured within 72 h post-exposure [e.g., 19,21,26,66]. However, the current study clearly illustrates that a 72-h assessment does not reflect sublethal effects of the treatment and the resulting long-term declines in survival. For instance, at 72 h post-exposure, survival at 63 d was 30% for both ice entrapment treatments but, after one-month mortality, was 100%. A 30-day timeframe post stress exposure is clearly a more ecologically relevant measure of survival but, crucially for polar species (with very long life cycles), this still misses development to subsequent life stages and any measure of fecundity (see also [70]).

These data suggest that, whilst winter microhabitat temperatures are cold enough for long enough to result in ice entrapment, this is clearly not the predominant experience of overwintering larvae given that the midge is still highly abundant and thriving on Signy Island [61]. It is possible that larvae avoid prolonged ice entrapment through microhabitat choice and, like *B. antarctica* or the mite *A. antarcticus*, will seek drier microhabitats to avoid inoculative freezing [71,72]. Both Bartlett et al. [17] and Hughes and Worland [67] posited that the patchy water content of Signy substrates drove patchiness in *E. murphyi* distributions in the studied summer seasons, so it is possible that similar habitat choices are made in the selection of overwintering sites. Any short-term flooding and freezing events that do entrap *E. murphyi* larvae, such as the freeze-thaw cycles of summer and the shoulder seasons [1], are not likely to result in significant mortality. 

During summer, it is possible for habitats on Signy to experience prolonged waterlogging as a result of snow melt, potentially leaving the terrestrial fauna exposed to anoxic conditions. In their study of the ability of *E. murphyi* to survive in a submerged environment, Everatt et al. [51] found that the midge could respire in water and was able to survive submergence for up to 28 days. Eltinsky et al. [72] also reported that *B. antarctica* was able to tolerate submergence in field water for up to 10 d, although capacity to respire was not studied. In the current study, nearly 50% of larvae survived for a further month following 28 days of submergence in FW (Figure 5). However, survival following 42 days submergence declined rapidly, and only a very small number of individuals survived just a few days after 63 days of submergence. The ability to tolerate submergence for up to 28 d, possibly longer, means that spring melt or rainfall which saturates the moss banks are unlikely to prove detrimental to the population in its current location on a slope with good drainage [71,73]. However, as the polar regions are affected by climate change, precipitation events and, therefore, habitat flooding are expected to increase [1,74]. 

### Implications for E. Murphyi as an Invasive Species

The ability to survive prolonged submergence does imply flexibility in habitat choice; in its native range on South Georgia, *E. murphyi* is found on the edge of streams [53], and currently, the midge is resident on Signy in well-drained moss banks. Survival up to 28 days fully submerged suggests that the species could establish in less steep ground that experiences periods of standing water. With a physiology similar to that of *B. antarctica*, Everatt et al. [19] suggested that there was no thermal limitation on *E. murphyi’s* ability to colonize areas that extend to a similar latitude, with the *B. antarctica* range extending to 68 °S [75]. Findings here further support this suggestion, particularly as all instars, and even the eggs have the physiological capacity to survive temperatures found at much higher latitudes than Signy; the LLTs of all larval stages and eggs are within the minimum winter recorded ground temperature for Anchorage Island at 68 °S [1]. 

## 5. Conclusions

The invasive midge *E. murphyi* switches cold-tolerance strategies within its life cycle. Larval instars are all freeze-tolerant, increasing in cold-hardiness with maturity. Eggs, however, appear to be freeze-avoiding with a much lower SCP than all the other life stages. Adults are the least cold-tolerant life stage and are perhaps best classified as chill-tolerant or chill-susceptible, much like adults of the related *B. antarctica*. All juvenile stages have the physiological capacity to survive microhabitat temperatures typically experienced during winter on Signy Island. While long-term (> 45 d) ice entrapment and submergence is lethal to *E. murphyi*, such prolonged periods of anoxia are unlikely to be experienced on Signy under the present conditions. The success of this introduced species on Signy is certainly partly the result of a preadapted physiology but is also supported by having access to buffered microhabitat conditions during winter extreme events. These features suggest a capacity for *E. murphyi* to both extend its distribution on Signy Island, as well as colonize areas further south. 

## Figures and Tables

**Table 1:** Results of two-way ANOVA with Tukey’s multiple comparisons for the effect of life stage on temperature survival (−5, −10, −15 and −20 °C) against the control (+5 °C). Listed with the significant drivers of the life-stage differences in response to temperature, that being the higher cold tolerance of eggs compared to larval instars. In each instance, the results returned DFn and DFd as 1 and 1490, respectively, but with variable F values (shown). No applicable significance below −20 °C, as all life-stages recorded 100% mortality.

Tukey’s Multiple Comparisons Test (°C)	Overall Life-Stage Influence *p*-Value	F Value (1, 1490)	Significant Life-Stage Interactions
5 vs. −5	< 0.0001	82.31	Eggs vs. all larvae ***p* < 0.0001**
5 vs. −10	< 0.0001	43.13	Eggs vs. L2, 3 and 4 ***p* < 0.001** L1 vs. L2 ***p* = 0.02**
5 vs. −15	< 0.001	52.29	Eggs vs. all larvae ***p* < 0.01**
5 vs. −20	< 0.001	4.28	Eggs vs. all larvae (eggs only survivors) ***p* < 0.001**

**Table 2:** Results of Kruskal-Wallis test with Dunn’s multiple comparisons for the influence of ice entrapment (NA/A) or submergence (C1) on L4 survival against the soil control (C2). Overall interaction of conditions with control: 28 d, H = 5.5, *p* = 0.13; 42 d, H = 13.2, *p* = 0.004 and 63 d, H = 18.3, *p* < 0.001.

Exposure (days)	Dunn’s Multiple Comparisons Test	Mean Rank Diff.	Adjusted *p*-Value
**28**	C2 vs. C1	9.143	0.109
	C2 vs. NA	0.8571	>0.999
	C2 vs. A	1.714	>0.999
**42**	C2 vs. C1	14.5	**0.002**
	C2 vs. NA	1.643	>0.999
	C2 vs. A	4.143	>0.999
**63**	C2 vs. C1	18.21	**<0.0001**
	C2 vs. NA	11.43	**0.026**
	C2 vs. A	12.36	**0.013**
